# Genomic Comparative Analysis of *Cordyceps pseudotenuipes* with Other Species from *Cordyceps*

**DOI:** 10.3390/metabo12090844

**Published:** 2022-09-08

**Authors:** Yingling Lu, Yi Wang, Xiaolong Yuan, Ou Huang, Quanying Dong, Dandan Li, Shujin Ding, Fuxian Ma, Hong Yu

**Affiliations:** 1 Yunnan Herbal Laboratory, College of Ecology and Environmental Sciences, Yunnan University, Kunming 650504, China; 2 School of Life Science, Yunnan University, Kunming 650504, China; 3 Laboratory of Forest Plant Cultivation and Utilization, The Key Laboratory of Rare and Endangered Forest Plants of State Forestry Administration, Yunnan Academy of Forestry and Grassland, Kunming 650201, China; 4 College of Forestry, Southwest Forestry University, Kunming 650224, China

**Keywords:** *Cordyceps*, whole-genome sequence, secondary metabolite, biosynthesis gene cluster

## Abstract

The whole genome of *Cordyceps pseudotenuipes* was sequenced, annotated, and compared with three related species to characterize the genome. The antibiotics and Secondary Metabolites Analysis Shell (antiSMASH) and local BLAST analysis were used to explore the secondary metabolites (SMs) and biosynthesis gene clusters (BGCs) of the genus *Cordyceps*. The genome-wide basic characteristics of *C. pseudotenuipes*, *C. tenuipes*, *C. cicadae*, and *C. militaris* revealed unequal genome size, with *C. cicadae* as the largest (34.11 Mb), followed by *C. militaris* (32.27 Mb). However, the total gene lengths of *C. pseudotenuipes* and *C. tenuipes* were similar (30.1 Mb and 30.06 Mb). The GC contents of *C. pseudotenuipes*, *C. tenuipes*, *C. cicadae*, and *C. militaris* genomes differed slightly (51.40% to 54.11%). AntiSMASH and local BLAST analysis showed that *C. pseudotenuipes*, *C. tenuipes*, *C. cicadae*, and *C. militaris* had 31, 28, 31, and 29 putative SM BGCs, respectively. The SM BGCs contained different quantities of polyketide synthetase (PKS), nonribosomal peptide synthetase (NRPS), terpene, hybrid PKS + NRPS, and hybrid NRPS + Other. Moreover, *C. pseudotenuipes*, *C. tenuipes*, *C. cicadae*, and *C. militaris* had BGCs for the synthesis of dimethylcoprogen. *C. pseudotenuipes*, *C. tenuipes*, and *C. cicadae* had BGCs for the synthesis of leucinostatin A/B, neosartorin, dimethylcoprogen, wortmanamide A/B, and beauvericin. In addition, the SM BGCs unique to *C. pseudotenuipes* were clavaric acid, communesin, and deoxynivalenol. Synteny analysis indicated that the scaffolds where the SM BGC was located were divided into more than 70 collinear blocks, and there might be rearrangements. Altogether, these findings improved our understanding of the molecular biology of the genus *Cordyceps* and will facilitate the discovery of new biologically active SMs from the genus *Cordyceps* using heterologous expression and gene knockdown methods.

## 1. Introduction

**Figure 1:**
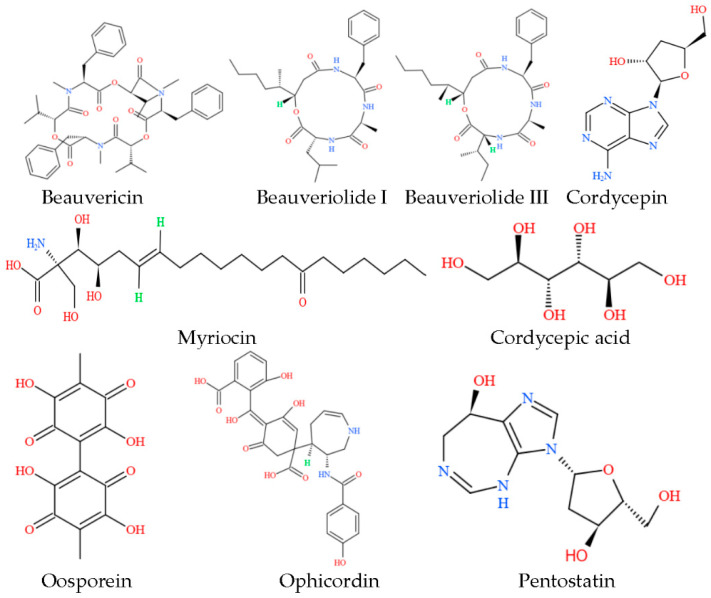
Structures of secondary metabolites from *Cordyceps*.

*Cordyceps pseudotenuipes* H. Yu, Q. Y. Dong, and Y. Wang is a new fungal species that was published in April 2022 [1]. The species was named after its morphological similarity to *C. tenuipes* (Peck) Kepler, B. Shrestha, and Spatafora. Taxonomically, this species was related to *C. tenuipes* and similar groups, including *C. cicadae* (Miq) Massee and *C. militaris* Fr. [1,2,3]. Genus *Cordyceps* had many bioactive components, including cordycepin [4], cordyceps polysaccharides [5], alkaloids [6], cordycepic acids [7], pentostatin [8], ophicordin [9], beauvericin [10], myriocin [11], beauveriolide [12], and oosporein [13] (Figure 1). These bioactive components function in various ways. For example, cordycepin was involved in synthesizing nucleic acid, platelet aggregation, and cell apoptosis and exhibits anticancer activity by incorporating it into RNA synthesis to break it off [14,15,16]. Moreover, cordycepin also treated mice infected with *Trypanosoma evansi* [17]. Cordycepin and pentostatin were used as chemotherapeutic drugs for leukemia [18], and combining both components increased their antitumor effect by over three times [19]. Oosporein, another bioactive component, was a polyketone compound with antibiotic, antiviral, antifungal, and insecticidal activities [20].

Genome mining is a computational method that automatically detects and annotates biosynthetic gene clusters (BGCs) from genomic data [21]. Today, the development of genome sequences, such as the identification and characterization of new compounds and metabolic engineering, have increased the range of applications of genome mining [22]. For instance, the antibiotics and Secondary Metabolites Analysis Shell (antiSMASH) web server was a stand-alone tool that was widely used for identifying and analyzing secondary metabolites (SMs) and BGCs in bacterial and fungal genomes [23]. The antiSMASH web server contributed significantly to microbial genome mining for new SM discoveries [24]. Wang et al. showed that the *C. militaris cns1*-*cns3* gene cluster produces cordycepin and pentostatin, where *cns1* and *cns2* were required for synthesizing cordycepin, and *cns3* was essential for pentostatin synthesis, elucidated by their biosynthetic pathways [25]. Moreover, nonribosomal peptide synthetase (NRPS; T-C-A-T-C-C-A-T-C-A-T-C-C), polyketide synthetase (PKS; KS-AT-DH-MT-ER-KR-ACP), acyltransferase, and ATP-dependent long chain fatty acyl-CoA synthetase in the genome of *C. militaris* catalyzes the synthesis of beauveriolide. The compound was generated by heterologous expression in *Aspergillus nidulans* (Eidam) G. Winter [26].

Whole-genome analysis of *C. militaris* CM01 revealed a cryptic gene cluster of encoding highly reducing polyketide synthase (HR-PKS), enol reductase (ER), and cytochrome P450. In *A. nidulans*, heterologous expression of the cryptic gene cluster produced two novel polyketide compounds, cordypyrone A and B [27]. From the bioinformatic analysis of the whole-genome sequence of *C. militaris* CM01’s two strains, 1630 and DSM 1153, Wang et al. identified two NRPS genes, one PKS, and a hybrid cluster. They predicted the structural characteristics of their potential products [28]. In order to further explore the biosynthetic potential of strain *C. pseudotenuipes* SM, its whole genome was sequenced and analyzed, while its whole genome data were compared with those of *C. tenuipes*, *C. militaris*, and *C. cicadae,* as well as for gene mining studies. The results showed that *C. pseudotenuipes*, *C. tenuipes*, *C. militaris*, and *C. cicadae* all contained different amounts of BGCs, including PKS, NRPS, terpene synthase (TPS), hybrid PKS + NRPS, and hybrid NRPS + Other.

## 2. Materials and Methods

### 2.1. Test Materials

*C. tenuipes*, *C. cicadae*, *C. militaris*, and *C. pseudotenuipes* strains were collected from Sapa, Vietnam; Dimalo village, Bangdang Township, Gongshan County, Yunnan Province; Zixi mountain, Zixi Town, Chuxiong City, Yunnan Province; and Yeyahu Forest Park, Kunming City, Yunnan Province. The voucher specimens were stored in Yunnan Herbal Herbarium (YHH) of Yunnan University, and the isolated strains were stored in Yunnan Fungal Culture Collection (YFCC) of Yunnan University. The whole-genome data of *C. tenuipes* were obtained from Yunnan Herbal Laboratory, College of Ecology and Environmental Sciences, Yunnan University. The genome-wide data of *C. cicadae* and *C. militaris* were obtained from NCBI (https://www.ncbi.nlm.nih.gov/ (accessed on 3 March 2022)), accession numbers ASM296887v1 and GCA_000225605.1, respectively.

### 2.2. Strain Culture

*C. pseudotenuipes* was cultured on an MY solid medium (21 g/L Yeast Malt Broth, 15 g/L Agar Powder, 1 L H_2_O) at 28 °C for 10 days. The mycelium was transferred to fresh MY liquid medium (21 g/L Yeast Malt Broth, 1 L H_2_O) at 28 °C at 150 r/min. After 8 days of culture, mycelia were collected, and three biological replicates of each sample were stored in a refrigerator at −80 °C until they were transported on dry ice to Personalbio (Shanghai, China) for high-throughput sequencing.

### 2.3. Genome Sequencing and Assembly

We used the Illumina NovaSeq 2000 platform (Illumina, CA, USA) to construct the *C. pseudotenuipes* gene library with 400 bp insert fragments. Raw data were processed using FastQC, 3′-terminal DNA junction was decontaminated using AdapterRemoval (version 2) [29], and Soapec (V2.0) software was used to perform quality correction on all reads based on the KMER frequency that the KMER used for correction was set to 17 to obtain high-quality adaptor-free genome sequences. The data were assembled de novo to construct the contigs and scaffolds. The obtained contigs and scaffolds were corrected using pilon v1.18 [30] software.

### 2.4. Gene Prediction and Annotation

Gene prediction was performed using homology, SNAP, and Augustus software. The predicted genes were annotated through BLAST searches against existing databases, including NCBI non-redundant protein sequences (Nr), Swiss prot, Kyoto Encyclopedia of Genes and Genomes (KEGG), Evolutionary Genealogy of Genes: Non-supervised Orthologous Groups (EggNOg), Pathogen–Host Interactions Database (PHI), and Carbohydrate-Active Enzymes (CAZy).

### 2.5. Secondary Metabolite Biosynthesis Gene Cluster Analysis

Gene cluster predictions for *C. tenuipes*, *C. cicadae*, *C. militaris*, and *C. pseudotenuipes* scaffolds were performed using the antiSMASH (https://antismash.secondarymetabolites.org/ (accessed on 27 May 2022)) online program. AntiSMASH detected scaffolds with gene clusters by using *C. militaris* as a parameter. The FGENESH (www.softberry.com/ (accessed on 28 May 2022)) online program predicted gene structures, and the PKS/NRPS online program (nrps.igs.umaryland.edu/ (accessed on 1 June 2022)) determined the gene clusters in contigs containing genes with NRPS/PKS structural domains. Meanwhile, the BLAST analysis (https://blast.ncbi.nlm.nih.gov/ (accessed on 8 June 2022)) was used for protein structure comparison to identify contigs harboring the NRPS/PKS gene.

### 2.6. Cluster Analysis

Known NRPS, PKS, and hybrid PKS-NRPS protein sequences were downloaded from NCBI and compared with protein sequences from this study using the Clustal W program of the MEGA5.0 software of IQ-TREE. The IQ-TREE web server is fast and accurately generates phylogenetic trees using the maximum likelihood method (http://iqtree.cibiv.univie.ac.at/ (accessed on 25 June 2022)). The analysis involved 1000 bootstraps using default parameters for constructing the cluster tree.

### 2.7. Synteny Analysis

Scaffolds of *C. cicadae*, *C. militaris*, *C. pseudotenuipes*, and *C. tenuipes* genome containing BGC of SM were analyzed, combining MAUVE v2.4.0 according to the assembly order for collinearity analysis.

## 3. Results

### 3.1. Basic Features of the C. pseudotenuipes Genome

#### 3.1.1. Genome Sequencing and Assembly

A total of 34,711,656 raw reads were obtained by Illumina sequencing (Illumina Ina, San Diego, CA, USA), yielding 34,001,494 HQ reads. The whole genome of *C. pseudotenuipes* was 30.1 Mb, containing 645 contigs and 527 scaffolds with an N50 of 131,856 bp and 54.11% GC content. The 8705 protein-coding genes with 13.97 Mb gene length were predicted, the average sequence length 1605.2 bp and the longest contig measured 0.41 Mb (Table 1). For non-coding RNA, we used tRNAscan, RNAmmer, and rfam_scan to predict 124 tRNA secondary structures, 31 rRNA, and 37 snRNA, respectively.

#### 3.1.2. *C. pseudotenuipes* Genome Annotation

**Figure 2:**
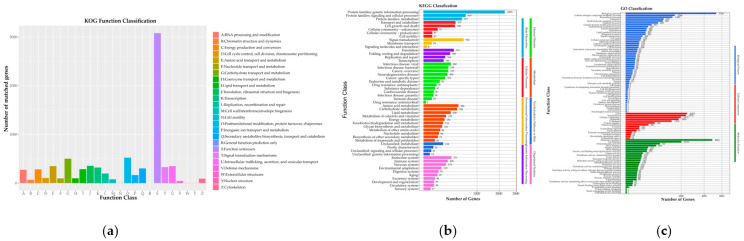
Functional annotation of *C. pseudotenuipes* gene-encoding proteins in the (**a**) Evolutionary Genealogy of Genes: Non-supervised Orthologous Groups of proteins (EggNOg), (**b**) Kyoto Encyclopedia of Genes and Genomes (KEGG), and (**c**) Gene Ontology (GO) databases.

Similarly, analysis of the 8705 non-redundant *C. pseudotenuipes* genes on publicly available protein sequence databases yielded varying results. The NCBI Nr (8596 genes) had 98.75%, Swiss-Prot (6206) had 71.29%, KEGG 3614 had 41.52%, GO (3614 genes) had 41.52%, EggNOg (7877) had 90.49%, Cytochrome P450 (CYP) (8480) had 97.42%, and Transporter Classification Database (TCDB) (1358) had 15.60% similarity to the 8705 predicted proteins (Table 1). The EggNOg database showed that most of the predicted genes were functionally associated with “Function unknown” (3079), “Posttranslational modification, protein turnover, chaperones”, and “Carbohydrate transport and metabolism” in that order (Figure 2a). Nonetheless, the wide variety of posttranslational events and carbohydrate metabolism suggested that they improved regulatory protein bioactivity and energy conversion efficiency. The KEGG functional classification revealed protein families for genetic information processing (2295), signaling and cellular processing (619), and signal transduction (562) (Figure 2b). Moreover, the rich diversity of genetic information and signaling proteins might facilitate more efficient information exchange and secondary metabolism. GO annotation indicated biological process (5316), cellular nitrogen compound metabolic process (1566), and biosynthetic process (1425) as the enriched terms from biological processes. Furthermore, the annotation revealed molecular function (4861), ion binding (2016), and oxidoreductase activity (767) from molecular function, and cell (2453), intracellular (2352), and cellular component (1972) from the cellular component (Figure 2c). *C. pseudotenuipes* was a wild strain; thus, many metabolic genes might be involved in signal transduction.

#### 3.1.3. *C. pseudotenuipes* Additional Annotation

##### Pathogen Host Interactions (PHI)

**Figure 3:**
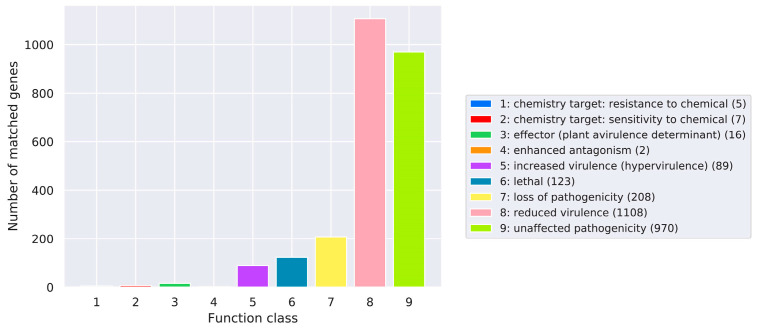
Distribution of the mutation types in the pathogen-PHI phenotype of *C. pseudotenuipes*.

Pathogen and host-interaction database (PHI-base), mainly from fungi-, oomycete-, and bacterial pathogen-infected hosts, included animals, plants, fungi, and insects [31]. Thus, the amino acid sequences of *C. pseudotenuipes* were aligned to obtain annotated results from the PHI-base (Figure 3). *C. pseudotenuipes* contained genes for reduced virulence (1108), unaffected pathogenicity (970), loss of pathogenicity (208), lethal (123), increased virulence (hypervirulence) (89), effector (plant avirulence determinant) (16), sensitivity to chemical (7), resistance to chemical (5), and enhanced antagonism (2). The results showed that the main annotated genes were for reduced virulence and unaffected pathogenicity, indicating that *C. pseudotenuipes* was a mildly pathogenic strain.

##### Carbohydrate Genes

**Figure 4:**
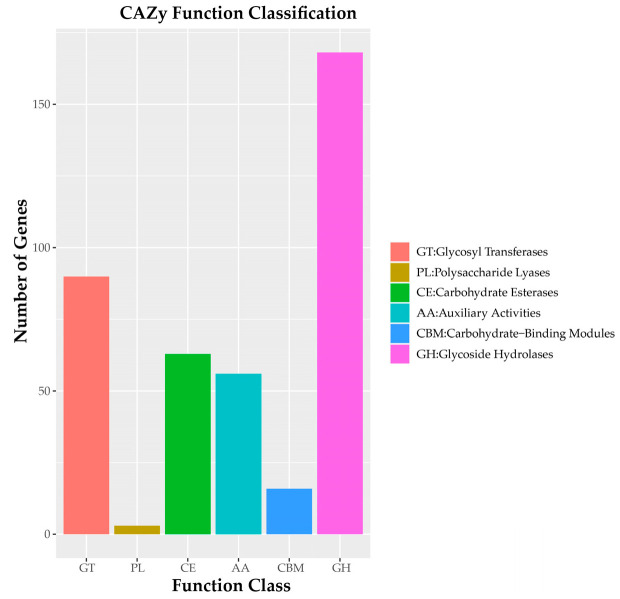
CAZy functional classification chart of *C. pseudotenuipes*.

Analysis revealed 396 gene-encoding carbohydrate-active enzymes (CAZy) in *C. pseudotenuipes*, including 168 glycoside hydrolases (GHs) and 63 carbohydrate esterases (CEs), 56 auxiliary activities (AAs), 16 carbohydrate-binding modules (CBMs), and 3 polysaccharide lyases (PLs) (Figure 4). CAZy catalyzed the assembly and breakdown of glycans and glycoconjugates [32]. Moreover, CAZy was a database of carbohydrate-active and carbohydrate enzymes [33]. It was suggested that *C. pseudotenuipes* should possibly capture more energy and decompose complex carbohydrates.

### 3.2. Characteristics of C. pseudotenuipes, C. tenuipes, C. cicadae, and C. militaris Genomes

The genome of *C. pseudotenuipes* was characterized and compared with its three close taxa (*C. tenuipes*, *C. cicadae,* and *C. militaris*) (Table 2). *C. cicadae* was the largest (34.11 Mb), followed by *C. militaris* (32.27 Mb), *C. pseudotenuipes* (30.1 Mb), and *C. tenuipes* (30.06 Mb). Moreover, *C. cicadae* had the highest number of scaffolds (595), *C. pseudotenuipes* and *C. cicadae* contained an equal number of scaffolds (527), *C. tenuipes* contained 285 scaffolds, and *C. militaris* had the lowest scaffold number (32). The genome containing the highest number of contigs was *C. cicadae* (1799), followed by *C. pseudotenuipes* (645), *C. militaris* (597), and *C. tenuipes* (384). However, the GC contents of *C. pseudotenuipes, C. tenuipes*, *C. cicadae*, and *C. militaris* were 54.11%, 53.72%, 52.70%, and 51.40%, respectively.

### 3.3. Analysis of Secondary Metabolite Biosynthesis Gene Cluster

AntiSMASH and local BLAST analyses showed that *C. pseudotenuipes* and *C. cicadae* had the same putative SM BGCs (31), followed by *C. militaris* (29) and *C. tenuipes* (28) (Tables S1 and S2). The SM BGCs presumably contained the highest number of *C. pseudotenuipes* NRPSs (16), followed by *C. cicadae* (15), *C. tenuipes* (13), and *C. militaris* (9). *C. militaris* had six PKS + NRPS hybrids, *C. cicadae* had five, and both *C. pseudotenuipes* and *C. tenuipes* had four. *C. tenuipes* contained the majority of NRPS + Other hybrids (3), *C. cicadae* and *C. militaris* had two, and *C. pseudotenuipes* had one. *C. pseudotenuipes*, *C. cicadae*, *C. militaris,* and *C. tenuipes* contained decreasing amounts of encoding TPS. *C. pseudotenuipes* (1), *C. cicadae* (1), *C. militaris* (2), and *C. tenuipes* (2) had similar numbers of other biosynthetic genes. *C. pseudotenuipes*, *C. tenuipes,* and *C. cicadae* had the same PKSs (5), while *C. militaris* had the highest number (8). The *C. pseudotenuipes* genome had five PKSs, including three HR-PKSs, two partially reducing (PR) PKSs, and one non-reducing (NR) PKS. The five PKSs obtained from the *C. tenuipes* genome included three NR-PKSs, one HR-PKS, and one PR-NRPS. The genome of *C. cicadae* had five PKSs, including two NR-PKSs, two HR-PKSs and one PR-NRPS. The eight PKSs retrieved from the *C. militaris* genome included five HR-PKSs, two PR-NRPSs, and one NR-PKS. These results showed that the number and type of SM BGCs obtained differed among species of the same genus.

The predicted BGCs showed different levels of genetic homology to known clusters in the MIBiG database, with *C. pseudotenuipes* having the highest homology (37.93%), followed by *C. tenuipes* (28.57%) and *C. cicadae* (25.81%). The BGCs predicted from *C. pseudotenuipes*, *C. tenuipes*, *C. cicadae*, and *C. militaris* genomes potentially synthesize dimethylcoprogen. In contrast, the BGCs predicted from *C. pseudotenuipes*, *C. tenuipes*, and *C. cicadae* predicted biosynthesis genes that catalyze the synthesis of leucinostatin A/leucinostatin B, neosartorin, dimethylcoprogen, wortmanamide A/wortmanamide B, and beauvericin. The antiSMASH and local BLAST analyses showed that only *C. tenuipes*, *C. cicadae*, and *C. militaris* potentially produce viriditoxin, while *C. tenuipes* and *C. cicadae* potentially produce trichodiene-11-one. Moreover, *C. pseudotenuipes*, *C. cicadae*, and *C. militaris* possibly catalyzed the synthesis of squalestatin S1; *C. pseudotenuipes* and *C. cicadae* also had BGCs for ilicicolin H synthesis, while *C. pseudotenuipes* and *C. tenuipes* were presumed responsible for epichloenin A synthesis. AntiSMASH analysis showed that ferrichrome was unique to *C. cicadae*, clavaric acid, communesin, and deoxynivalenol were unique to *C. pseudotenuipes*, and phomasetin, fumosorinone, and 1-nonadecene/(14z)-1,14-nonadecadiene were unique to *C. militaris*. Furthermore, similar species of the same genus had different types and quantities of BGCs and catalytically synthesized compounds.

**Figure 5:**
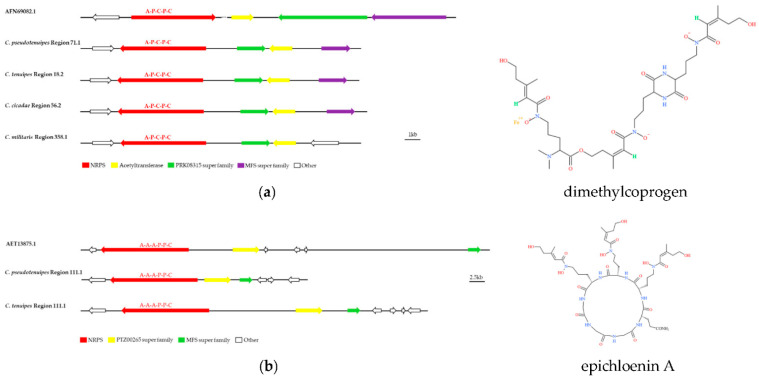
Comparison of BGC constituents in *C. pseudotenuipes*, *C. tenuipes*, *C. militaris,* and *C. cicadae* with identified BGCs for putative biosynthesis of dimethylcoprogen (**a**) and epichloenin A (**b**). The number after the region and the number before the decimal point represent the scaffold, and the number after the decimal point represents the gene cluster.

Several BGCs of *C. pseudotenuipes*, *C. tenuipes,* and *C. cicadae* were 100% similar to MIBiG sequences. Dimethylcodogen was siderophores, produced by the *Alternaria* species to obtain extracellular iron [34,35]. The predicted region 358.1 of *C. militaris*, region 71.1 of *C. pseudotenuipes*, region 56.2 of *C. cicadae*, and region 18.2 of *C. tenuipes* might be responsible for dimethylcodogen biosynthesis (Figure 5a). Region 111.1 of *C. pseudotenuipes* and region 34.1 of *C. tenuipes* were responsible for epichloenin A biosynthesis (Figure 5b), and region 2.2 of *C. pseudotenuipes* was responsible for clavaric acid biosynthesis. In contrast, *C. cicadae* and *C. militaris* lacked the potential to synthesize epichloenin A. The enzymes involved in the synthesis of dimethylcoprogen and epichloenin A were NRPS, and the enzyme involved in the synthesis of clavaric acid was terpene. A local BLAST comparison demonstrated that dimethylcoprogen synthesis required enzymes with the A-P-C-P-C structural domain, acetyltransferase, PRK08315 superfamily, and MFS superfamily. Moreover, epichloenin A synthesis required NRPS (A-A-A-P-C), PTZ00265 superfamily, and MFS superfamily.

**Figure 6:**
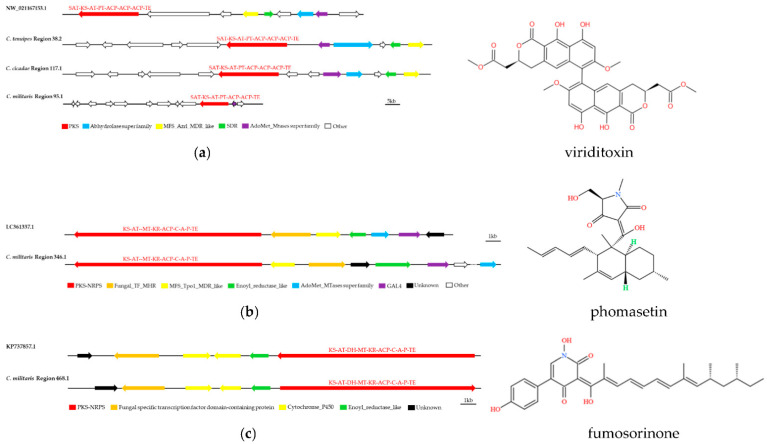
Comparison of biosynthesis of putative viriditoxin (**a**), phomasetin (**b**), and fumosorinone (**c**) biosynthetic gene clusters. The number after the region and the number before the decimal point represent the scaffold, and the number after the decimal point represents the gene cluster.

The five main biosynthetic genes in the putative SM BGCs of *C. pseudotenuipes*, *C. tenuipes*, *C. militaris*, and *C. cicadae* were similar to known gene clusters in the MIBiG database (ranging from 40% to 85%). Region 78.1 of *C. pseudotenuipes*, region 94.1 of *C. cicadae*, and region 426.1 of *C. militaris* are for squalestatin S1 synthesis. Region 38.2 of *C. tenuipes*, region 117.1 of *C. cicadae*, and region 95.1 of *C. militaris* probably synthesized viriditoxin, while region 1.2 of *C. cicadae* and region 346.1 and region 468.1 of *C. militaris* synthesized ferrichrome, phomasetin, and fumosorinone, respectively. The results showed that *C. pseudotenuipes* lacked the potential to synthesize viriditoxin and that catalytic synthesis of viriditoxin required several additional enzymes besides the core enzyme PKS (SAT-KS-AT-PT-ACP-ACP-ACP-TE). The additional enzymes included the Abhydrolase superfamily, MFS_Azrl_MDR_like, SDR, AdoMet_Mtases superfamily, and others of unknown function (Figure 6a). LC361337.1 synthesized phomasetin (Figure 6b). Furthermore, the core gene structural domain (KS-AT-MT-KR-ACP-C-A-P-TE) and modifier genes in the *C. militaris* region 346.1 were similar to LC361337.1. The location and orientation of the modifier genes differed, and the remaining three species lacked the potential to synthesize phomasetin. These results revealed that *C. militaris* region 468.1 was similar to the gene cluster synthesizing fumosorinone (Figure 6c). Both regions had the KS-AT-MT-KR-ACP-C-A-P-TE core structural domain, the fungal-specific transcription factor domain-containing protein, cytochrome P450, and enoyl-reductase modifier genes, indicating that the positions and directions of functional enzymes differed between species.

**Figure 7:**
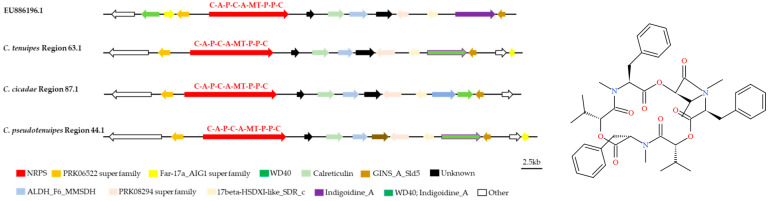
Comparison of putative beauvericin biosynthetic gene clusters and structure. The number after the region and the number before the decimal point represent the scaffold, and the number after the decimal point represents the gene cluster.

The antiSMASH and local BLAST analyses showed that the whole genomes of *C. tenuipes*, *C. cicadae*, and *C. pseudotenuipes* contained gene clusters that were highly similar to those responsible for the synthesis of Beauvericin (BEA) BGCs (GenBank: EU886196.1) (Figure 7). The putative NRPS from the *C. tenuipes* region 63.1, *C. cicadae* region 87.1, and *C. pseudotenuipes* region 44.1 had a C-A-P-C-A-MT-P-P-C structural domain, with precursors initiated and terminated by condensed structural domains. The NRPS also required several modifier genes, including the putative PRK06522, Far-17a_AIG1, and PRK08294 superfamilies, WD40, calreticulin, ALDH_F6_MMSDH, 17beta-HSDXI-like_ SDR_c, Indigoidine_A, and GINS_A_Sld5, although *C. cicadae* region 87.1 lacked the Far-17a_AIG1 superfamily. The high similarity of the BEA BGC between *Cordyceps* and *Beauveria bassiana* suggested some degree of horizontal gene transfer between these genera and that gene loss/addition might occur between different species, even though the gene sequences varied in orientation and position.

**Figure 8:**
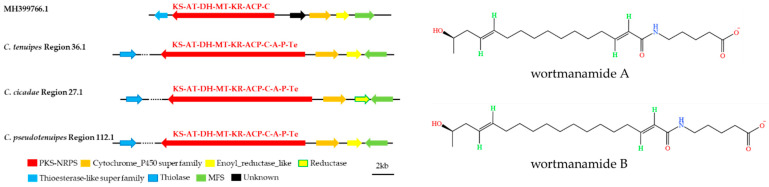
Comparison of putative wortmanamide A or wortmanamide B biosynthetic gene clusters and structure. The number after the region and the number before the decimal point represent the scaffold, and the number after the decimal point represents the gene cluster.

Furthermore, the gene clusters of *C. tenuipes* region 36.1, *C. cicadae* region 27.1, and *C. pseudotenuipes* region 112.1 resembled wortmanamide A or wortmanamide B BGC (Figure 8). Wortmanamide A or wortmanamide B was a reduced long-chain polyketide amidated by a specific ω-amino acid 5-aminopentanoic acid (5PA), initially found in *Talaromyces wortmannii*, a long-chain N-acylamide-like signaling lipid. A hybrid PKS-NRPS catalyzed synthesis with thioesterase and cytochrome P450 as the upstream modifier genes and enoyl_reductase and MFS as the downstream modifier gene synthesized wortmanamide A/B [36]. The hybrid PKS-NRPS structural domain of *C. tenuipes* region 36.1, *C. cicadae* region 27.1, and *C. pseudotenuipes* region 112.1 was KS-AT-DH-MT-KR-ACP-C-A-(P)-SDR. KS-AT-DH-MT-KR-ACP-C was the domain for catalytic wortmanamide A/B synthesis. The domains also had slightly different modifier genes. For instance, the modifier genes downstream of *C. tenuipes* region 36.1 was reductase, not enoyl_reductase, and thiolase, instead of thioesterase for *C. tenuipes* region 36.1, *C. cicadae* region 27.1, and *C. pseudotenuipes* region 112.1. Thus, *C. tenuipes* region 36.1, *C. cicadae* region 27.1, and *C. pseudotenuipes* region 112.1 might synthesize wortmanamide or its analogs.

### 3.4. Cluster Analysis

The clustering results of *Cordyceps* PKS and hybrid PKS-NRPS proteins with other fungal PKS and hybrid PKS-NRPS proteins showed that *C. militaris* region 346.1 clustered with *Pyrenochaetopsis* sp. (BBC43184.1), which catalyzed phomasetin biosynthesis (Figure S1). Moreover, *C. militaris* region 346.1 might catalyze the biosynthesis of phomasetin or its analogs. *C. militaris* region 95.1, *C. cicadae* region 117.1, and *C. tenuipes* region 38.2 clustered with the PKS protein and possibly catalyzes viriditoxin synthesis in *Paecilomyces variotii* (XP_0284818201). Moreover, *C. militaris* region 95.1, *C. cicadae* region 117.1, and *C. tenuipes* region 38.2 possibly catalyze viriditoxin or its analogs. *C. pseudotenuipes* region 112.1, *C. cicadae* region 27.1, *C. tenuipes* region 36.1, and *Talaromyces wortmannii* (QBC19710.1) were clustered on an independent branch that produced wortmanamide A/B. *C. pseudotenuipes* region 112.1, *C. cicadae* region 27.1, and *C. tenuipes* region 36.1 presumably catalyze the synthesis of wortmanamide A/B or its analogs. *C. militaris* region 468.1 clustered with *C. fumosorosea* (AKC54422.1), which catalyzed fumosorinone biosynthesis. Furthermore, *C. militaris* region 468.1 might catalyze the biosynthesis of fumosorinone or its analogs. *C. pseudotenuipes* region 71.1, *C. cicadae* region 56.2, and *C. tenuipes* region 18.2 clustered with *Alternaric alternata* (AFN69082.1) catalyzed dimethylcoprogen synthesis (Figure S2) and probably produced dimethylcoprogen or its analogs.

*C. pseudotenuipes* region 44.1, *C. cicadae* region 87.1, and *C. tenuipes* region 63.1 clustered on a separate branch from *Beauveria bassiana* (ACI30655.1), which catalyzed BEA biosynthesis, and the three regions presumably catalyze the biosynthesis of BEA or its analogs. Likewise, *C. pseudotenuipes* region 111.1 and *C. tenuipes* region 34.1 clustered on an independent branch with *Epichloe festucae* (AET13875.1), an *E. festucae* protein sequence that catalyzed epichloenin A biosynthesis. We hypothesized that *C. pseudotenuipes* region 111.1 and *C. tenuipes* region 34.1 might catalyze the synthesis of epichloenin A or its analogs.

### 3.5. Synteny Analysis

**Figure 9:**
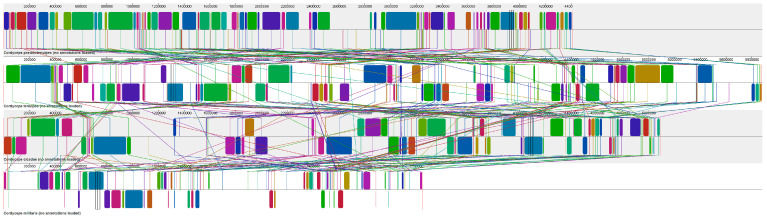
Scaffold containing secondary metabolite biosynthesis gene cluster used for synteny analysis.

The scaffolds containing the SM BGC in the genomes of *C. cicadae* (24), *C. militaris* (26), *C. pseudotenuipes* (27), and *C. tenuipes* (28) were subjected to synteny analysis. The scaffolds where the SM BGC were located were divided into more than 70 collinear blocks, and there may be rearrangements (Figure 9). From top to bottom, they were *C. pseudotenuipes*, *C. tenuipes*, *C. cicadae*, and *C. militaris*.

## 4. Discussion

This study showed that *Cordyceps* contains numerous bioactive components, such as cordycepin, cordycepic acids, polysaccharide, and cyclic peptides [9], that have various clinical health effects, including anticancer, antioxidant, anti-inflammatory, immunomodulatory, and antibacterial activities [37]. The recent, significant improvement in next-generation sequencing increased the speed and reduced the cost of sequencing genomes of fungi [38]. Thus, this study presented the basic genomic characteristics of *C. pseudotenuipes* and its approximate taxa, *C. tenuipes*, *C. cicadae*, *C. militaris*, *C. pseudotenuipes*, *C. tenuipes*, *C. cicadae*, and *C. militaris*, revealing differences in genome size, scaffold N50, contig N50, and GC content, although all the species belong to the same genus.

Fungal SMs were generally classified based on their building blocks into polyketides (PKs), such as lovastatin, nonribosomal polypeptides (NRPs), such as penicillin, and terpenes, such as gibberellin [26]. Nonribosomal polypeptides and PKs were the major SMs groups with significantly diverse structures and biological functions [39,40]. New genome sequences of dozens of filamentous fungi suggested that their potential for SM generation far exceeds previous estimates. Moreover, the addition of genome mining has significantly improved bioassay-guided natural product discovery. Yuan et al. found likely existing NRPs and PKs through genome mining of the genome of *C. militaris* [41]. In this study, whole-genome mining of *C. pseudotenuipes*, *C. tenuipes*, *C. cicadae*, and *C. militaris* revealed putative SM BGCs. AntiSMASH and local BLAST analyses revealed 29 putative SM BGCs in the *C. militaris* genome, including 9 NRPSs, 8 PKSs, 6 hybrids PKS + NRPS, 2 hybrids NRPS + Other, 2 terpene, and 2 other gene clusters. Elsewhere, Wang et al. sequenced and predicted the genome of *C. militaris* CM01 (GenBank: GCA_000225605.1) using the antiSMASH online tool and 27 clusters of SM biosynthesis genes, including 7 NRPS, 7 hybrid PKS-NRPS, 5 PKS, 4 terpenoid cyclase (TC), and 4 others [42]. The contrast between this study and Wang et al. was presumably due to different analysis methods. Wang et al. identified a gene cluster in the *C. militaris* genome that catalyzed beauveriolide synthesis using NRPS, PKS, acyltransferase, and ATP-dependent long-chain fatty acyl-coenzymes A synthase [26]. However, the antiSMASH and local BLAST analyses revealed that the modified genes in *C. militaris* regions 67.1 and 67.2 and between 67.1 and 67.2 were consistent with the gene cluster catalyzing the synthesis of the identified beauveriolide. *C. militaris* region 536.1 and its four downstream modifier genes were consistent with the gene clusters that catalyzed the synthesis of two novel polyketide compounds, cordypyrone A and B. [27]. Nonetheless, the wortmanamide BGC differed, suggesting that *C. tenuipes* region 36.1, *C. cicadae* region 27.1, and *C. pseudotenuipes* region 112.1 might catalyze the synthesis of wortmanamide or its analogs [36]. This study suggested that *C. cicadae* potentially catalyzed the synthesis of BEA or its analogs, confirming previous studies where BEA was isolable from *C. cicadae* [13]. Thus, the next step was to verify through heterologous expression or gene knockout whether *C. cicadae* region 87.1 and its modifier genes were BGCs that catalyze BEA biosynthesis.

Moreover, NRPS and PKS also appeared to hybridize PKS-NRPS or NRPS-PKS when catalyzing the synthesis of compounds. Chimeric genes produced chimeric compounds and biologically active hybrid products such as cyclopiazonic acid, pyranonigrin, and cytochalasin [42,43,44,45]. Seshime et al. functionally expressed the hybrid PKS-NRPS CpaA involved in cyclopiazonic acid biosynthesis in *A. flavus* [46]. Moreover, Nielsen et al. successfully constructed hybrid PKS-NRPS expressing functional cross-species in *A. nidulans*, indicating that a rational redesign of fungal natural product enzymes was feasible [47]. Theobald et al. also showed the origin of hybrid PKS-NRPS in *Aspergilli* [44], and Zhong et al. identified a hybrid PKS-NRPS gene cluster from a clonal population of the rice blast fungus *Magnaporthe oryzae* [48]. Tang et al. also identified a PKS-NRPS hybrid enzyme and ten pyranterreones products in *A. terreus* through genome mining [49]. Sigrist et al. studied cis/trans hybrid PKS-NRPS of nonlinear biosynthetic assembly Alpiniamided, suggesting that it might take place on two parallel assembly lines, the products of which were then linked together [50]. In that order, *C. pseudotenuipes*, *C. tenuipes*, *C. cicadae*, and *C. militaris* genomes analyzed in this study showed that the type and number of putative BGCs differ among species of the same genus. *C. pseudotenuipes*, *C. tenuipes*, *C. cicadae*, and *C. militaris* contained 4, 4, 5, and 6 hybrid PKS-NRPS, respectively. Thus, gene chimerism probably occurred in the PKS and NRPS of genus *Cordyceps* and catalyzed the synthesis of chimeric compounds. The antiSMASH and local BLAST results showed that horizontal gene transfer possibly occurs among these species of the same genus, explaining the variable direction and position of gene sequences. The next step is to validate the SM BGCs of *C. pseudotenuipes*, *C. tenuipes*, *C. cicadae*, and *C. militaris* through heterologous expression or gene knockdown.

The synteny analysis of scaffolds containing SM BGC In *C. pseudotenuipes*, *C. tenuipes*, *C. cicadae*, and *C. militaris* genome showed that the collinearity of *C. militaris* sequences was significantly different from that of *C. pseudotenuipes*, *C. tenuipes*, and *C. cicadae*. It was presumed that the classification of *C. militaris* was far from that of *C. pseudotenuipes*, *C. tenuipes*, and *C. cicadae*.

## 5. Conclusions

In order to deeply explore the SMs BGCs of the genus *Cordyceps*, the whole genome of *C. pseudotenuipes* was sequenced, annotated, and compared with three related species to characterize the genome. The high-quality whole-genome sequence of *C. pseudotenuipes* was obtained and extensively analyzed by gene prediction and annotation in this work. The results demonstrated that *C. pseudotenuipes* harbored abundant functional genes in regulatory protein bioactivity and conversion, signal transduction and metabolism, carbohydrate transport and metabolism, capturing energy, and decomposes carbohydrates.

The genome-wide basic characteristics of *C. pseudotenuipes*, *C. tenuipes*, *C. cicadae*, and *C. militaris* revealed unequal genome sizes and GC contents. AntiSMASH and local BLAST analyses showed that *C. pseudotenuipes*, *C. tenuipes*, *C. cicadae*, and *C. militaris* had different amounts and types of putative SM BGC. Presumably, only seven of the putative BGCS were highly similar to known gene clusters, indicating a great potential to generate other SMs. These findings opened the possibility of targeted genomic mining, such as gene knockdown, introduction or heterologous expression of microbial genes, promoter regulation, and mutation induction, to awaken the silenced BGC biosynthesis of more novel bioactive SMs for new drug research and development.

## Figures and Tables

**Table 1:** Genomic assembly and functional annotation of *C. pseudotenuipes* genome.

Item	Value	Item	Count	Percentage (%)
Total length (Mb)	30.1	NR	8596	98.75
Max length (bp)	413,323	SwissProt	6206	71.29
GC content (%)	54.11	KEGG	3614	41.52
Gene number	8705	GO	5861	62.33
Total gene number (bp)	13,973,781	EggNOg	7877	90.49
Average gene number (bp)	1605.2	P450	8480	97.42
Gene/Genome (%)	47.3169	TCDB	1358	15.60
Contigs	645			
Scaffolds	527			
Contigs N50	101,518			
Scaffolds N50	131,856			
Contigs N90	27,054			
Scaffolds N90	32,698			

**Table 2:** Characteristics of *C. pseudotenuipes*, *C. tenuipes*, *C. cicadae*, and *C. militaris* genomes.

Item	*C. pseudotenuipes* This Study	*C. tenuipes* This Study	*C. cicadae* ASM296887v1	*C. militaris* GCA_000225605.1
Contigs	645	384	1799	597
Scaffolds	527	285	595	32
Total length (Mb)	30.1	30.06	34.11	32.27
GC content (%)	54.11	53.72	52.70	51.40
Scaffold N50 (bp)	131,856	172,867	212,207	4,551,492
Contig N50 (bp)	101,518	140,681	47,316	10,818

## Data Availability

The data presented in this study are available in supplementary material. *C. pseudotenuipes* and *C. tenuipes* genome data are available from NCBI.

## Supplementary Materials

The following supporting information can be downloaded at: https://www.mdpi.com/article/10.3390/metabo12090844/s1, Table S1: Putative biosynthetic gene clusters (BGCs) coding for secondary metabolites in three *Cordyceps* species. Table S2: Overview of biosynthetic gene clusters in the genomes of the four studied fungi. Figure S1: Clustering of the PKS/hybrid PKS-NRPS and other related fungal PKS/hybrid PKS-NRPS proteins in the four *Cordyceps* species. Figure S2: Clustering of NRPS and other fungal NRPS proteins in the four species of *Cordyceps*.
