# Supplementary material for: Genomic Comparative Analysis of Cordyceps pseudotenuipes with Other Species from Cordyceps
Source: Metabolites. 2022 Sep 8;12(9):844. doi: 10.3390/metabo12090844 (PMC9505148; doi:10.3390/metabo12090844)
Supplement: Supplementary file 1 [file metabolites-12-00844-s001.zip › metabolites-1894742-supplementary.pdf]

Supplementary materials for

Genomic Comparative Analysis of *Cordyceps pseudotenuipes* with Other Species from *Cordyceps*

Yingling Lu [1,2,3], Yi Wang [3,*], Xiaolong Yuan [3], Ou Huang [1], Quanying Dong [1,2], Dandan Li [1], Shujin Ding [3,4], Fuxian Ma [3,4], and Hong Yu [1,*]

[1] Yunnan Herbal Laboratory, College of Ecology and Environmental Sciences, Yunnan University, Kunming 650504, China
[2] School of Life Science, Yunnan University, Kunming 650504, China
[3] Laboratory of Forest Plant Cultivation and Utilization, The Key Laboratory of Rare and Endangered Forest Plants of State Forestry Administration, Yunnan Academy of Forestry and Grassland, Kunming 650201, China
[4] College of Forestry, Southwest Forestry University, Kunming 650224, China
* Correspondence: wangyi@ynlky.org.cn (Y.W.); hongyu@ynu.edu.cn (H.Y.); Tel.: +186-8716-3524 (Y.W.); +137-0067-6633 (H.Y.)

Table S1. Putative biosynthetic gene clusters (BGCs) coding for secondary metabolites in three *Cordyceps* species.

Species	BGCs	Location	From	To	Domain	Type	Most similar known cluster (%)
C. pseudotenuipes	Region 5.1	Scaffold5.g87	285004	289464	A-P-C	NRPS	Unknown
	Region 16.1	Scaffold16.g49	147482	161065	A-A-A-P	NRPS	Leucinostatin A/B(10%)
	Region 36.1	Scaffold36.g47	48581	52612	A-P-T	NRPS	Unknown
	Region 40.1	Scaffold40.g3	8362	12614	A	NRPS	Unknown
	Region 44.1	Scaffold44.g36	115984	125787	C-A-P-C-A-MT-P-P-C	NRPS	Beauvericin(50%)
	Region 62.2	Scaffold62.g14	55895	60812	A-P-C	NRPS	Unknown
	Region 63.2	Scaffold63.g30	102232	106851	A-C	NRPS	Unknown
	Region 71.1	Scaffold71.g31	105741	111176	A-P-C-P-C	NRPS	Dimethylcoprogen(100%)
	Region 71.2	Scaffold71.g32	113201	115000	A	NRPS	Unknown

	Region 95.1	Scaffold95.g17	29739	36995	A-P-TE	NRPS	Unknown
	Region 110.1	Scaffold110.g13	29035	37080	A-TE	NRPS	Unknown
	Region 109.2	Scaffold109.g20	67996	82620	A-P-A-C-P-C-P-C	NRPS	Unknown
	Region 111.1	Scaffold111.g19	50319	64379	A-A-P-P-C	NRPS	Epichloenin(100%)
	Region 4.1	Scaffold4	327204	369543		NRPS	Unknown
	Region 14.1	Scaffold14	161344	205168		NRPS	Unknown
	Region 30.1	Scaffold30	177519	211854		NRPS	Unknown
	Region 2.1	Scaffold2.g62	202641	214348	A-P-KS-AT-ACP-TE-A-P-C	NRPS,T1PKS	Unknown
	Region 61.1	Scaffold61.g15	36494	50344	KS-AT-PT-DH-MT-KR-ACP-C-A-TE	NRPS,T1PKS	Ilicicolin H(33%)
	Region 69.1	Scaffold69.g8	20216	25110	C-A-KS-AT	NRPS,T1PKS	Unknown
	Region 112.1	Scaffold112.g12	48710	61703	KS-AT-DH-MT-KR-ACP-C-A-P-TE	NRPS,T1PKS	Wortmanamide A/B(33%)
	Region 203.1	Scaffold203.g11	35814	40761	DIT-A-P-TE	NRPS,Other	Unknown
	Region 38.1	Scaffold38.g13	56687	63629	SAT-KS-AT-PT-ACP-TE	NR-PKS	Neosartorin(15%)
	Region 62.1	Scaffold62.g12	48167	49246	ER	PR-PKS	Unknown
	Region 63.1	Scaffold63.g26	87191	94682	KS-AT-DH-Ser-KR	PR-PKS	Unknown
	Region 166.1	Scaffold166.g3	14199	21551	KS-AT-DH-MT-ER-KR	HR-PKS	Unknown
	Region 97.1	Scaffold97.g9	29739	36995	KS-AT-DH-ACP-ER-KR-ACP	HR-PKS	Communesin A-E 、 G-H(12%)
	Region 2.2	Scaffold2	346033	367916		Terpene	Clavaric acid(100%)
	Region 74.1	Scaffold74	71680	93695		Terpene	Unknown
	Region 78.1	Scaffold78	101894	117833		Terpene	Squalestatin S1(40%)
	Region 210.1	Scaffold210	24954	39266		Terpene	Deoxynivalenol(22%)
	Region 143.1	Scaffold143	55376	65837		Siderophore	Unknown
C. tenuipes	Region 1.1	Scaffold1.g65	215520	229109	A-P-C-A-P-A-P	NRPS	Leucinostatin A/B(10%)

Region 5.1	Scaffold5.g22	62602	76647	A-A-A-P-P-C	NRPS	Unknown
Region 12.1	Scaffold12.g82	261121	265367	A-P-C	NRPS	Unknown
Region 18.1	Scaffold18.g53	196659	198458	A	NRPS	Unknown
Region 18.2	Scaffold18.g54	200486	205921	A-P-C-A-P-C	NRPS	Dimethylcoprogen(100%)
Region 26.1	Scaffold26.g19	78329	82153	A-P-C	NRPS	Unknown
Region 34.1	Scaffold34.g22	62602	76647	A-A-A-P-P-C	NRPS	Epichloenin(100%)
Region 63.1	Scaffold63.g25	83070	92870	C-A-P-C-A-MT-P-P-C	NRPS	Beauvericin(50%)
Region 69.1	Scaffold69.g34	124018	138119	A-P-A-C-P-C-P-C	NRPS	Unknown
Region 58.1	Scaffold58.g11	39231	43584	A-C	NRPS	Unknown
Region 77.1	Scaffold77.g32	99102	101399	C-A	NRPS	Trichodiene-11-one(27%)
Region 39.1	Scaffold39.g43	121861	125925	A-P	NRPS	Unknown
Region 151.1	Scaffold151	9620	52727		NRPS	Unknown
Region 36.1	Scaffold36.g13	51646	64637	KS-AT-DH-MT-KR-P-C-A-P-TE	NRPS,T1PKS	Wortmanamide A/B(50%)
Region 58.2	Scaffold58.g14	55027	66873	KS-AT-DH-MT-KR-P-C-A-P-TE	NRPS,T1PKS	Unknown
Region 136.1	Scaffold136.g13	32646	45027	KS-AT-DH-MT-KR-P-C-A-TE	NRPS,T1PKS	Unknown
Region 180.1	Scaffold180.g8	38068	42699	A-PT-C	NRPS,T1PKS	Unknown
Region 1.2	Scaffold1.g127	425180	429046	A-P-NAD-TE	NRPS,Other	Unknown
Region 38.1	Scaffold38.g1	715	5665	DIT-A-P-TE	NRPS,Other	Unknown
Region 139.1	Scaffold139.g7	13380	19234	A-TE-Lipase	NRPS,Other	Unknown
Region 70.1	Scaffold70.g28	92209	103919	A-KS-AT-PT-P-TE	NR-PKS	Unknown
Region 15.1	Scaffold15.g81	247464	254376	SAT-KS-AT-PT-DH-ACP-ACP-TE	NR-PKS	Neosartorin(15%)
Region 38.2	Scaffold38.g60	192908	200141	SAT-KS-AT-PT-ACP-ACP-ACP-TE	NR-PKS	Viriditoxin(66%)
Region 154.1	Scaffold154.g4	7275	14757	KS-AT-DH-Ser-KR-ACP	PR-PKS	Unknown
Region 9.1	Scaffold9.g107	353166	360446	KS-AT-DH-Ser-ER-KR-ACP	HR-PKS	Unknown
Region 27.1	Scaffold27.g29	87786	88709	TE	Other	Unknown

	Region 59.1	Scaffold59	143002	153655		Siderophore	Unknown
	Region 84.1	Scaffold84	125344	140662		Terpene	Unknown
C. cicadae	Region 1.1	Scf00001.g35	119793	123659	A-P-TE	NRPS	Unknown
	Region 1.2	Scf00001.g99	327555	341271	A-A-P-A-P	NRPS	Ferrichrome(66%)
	Region 1.3	Scf00001.g211	685792	690038	A-P-C	NRPS	Unknown
	Region 6.1	Scf00006.g101	377154	381502	A-C	NRPS	Unknown
	Region 38.1	Scf00038.g38	113515	117570	A-P-T	NRPS	Unknown
	Region 39.1	Scf00039.g41	153658	157482	A-P-C	NRPS	Unknown
	Region 39.2	Scf00039.g66	217296	220238	A-P-TE	NRPS	Unknown
	Region 56.1	Scf00056.g6	21350	23149	A	NRPS	Unknown
	Region 56.2	Scf00056.g7	25525	30960	A-P-C-P-C	NRPS	Dimethylcoprogen(100%)
	Region 59.1	Scf00059.g30	88994	91273	A	NRPS	Unknown
	Region 59.2	Scf00059.g35	108003	109628	C	NRPS	Unknown
	Region 87.1	Scf00087.g8	19232	30142	C-A-P-C-A-MT-P-P-C	NRPS	Beauvericin(50%)
	Region 158.1	Scf00158.g12	29605	33008	A-TE	NRPS	Unknown
	Region 183.2	Scf00183.g9	25888	30606	A-C	NRPS	Unknown
	Region 222.1	Scf00222	1	27708		NRPS	Unknown
	Region 6.2	Scf00006.g105	403977	415814	KS-AT-DH-MT-KR-P-C-A-P-TE	NRPS,T1PKS	Unknown
	Region 27.1	Scf00027.g51	198137	211143	KS-AT-DH-MT-KR-ACP-C-A-P-TE	NRPS,T1PKS	Wortmanamide A/B(50%)
	Region 29.1	Scf00029.g74	252612	264375	A-P-KS-AT-ACP-TE-A-P-C	NRPS,T1PKS	Unknown
	Region 109.1	Scf00109.g6	9516	21901	KS-AT-DH-MT-KR-ACP-C-A-TE	NRPS,T1PKS	Ilicicolin H(33%)
	Region 159.1	Scf00159.g6	12558	24106	KS-AT-DH-ER-KR-P-C-A-P	NRPS,T1PKS	Unknown
	Region 53.1	Scf00053.g1	11323	19713	A-HMA-ZntA	NRPS,Other	Unknown
	Region 92.1	Scf00092.g21	58518	63474	DIT-Caic-P-TE	NRPS,Other	Unknown
	Region 12.1	Scf00012.g9	20169	27153	SAT-KS-AT-PT-DH-P-P-TE	NR-PKS	Neosartorin(15%)

	Region 117.1	Scf00117.g18	49853	57073	SAT-KS-AT-PT-ACP-ACP-ACP-TE	NR-PKS	Viriditoxin(66%)
	Region 183.1	Scf00183.g5	10103	17659	KS-AT-DH-KR-ACP	PR-PKS	Unknown
	Region 90.1	Scf00090.g14	49329	56498	KS-AT-DH-Ser-ER-KR-ACP	HR-PKS	Unknown
	Region 219.1	Scf00219.g3	12936	20664	KS-AT-DH-MT-ER-KR-P	HR-PKS	Leucinostatin A/B(10%)
	Region 40.1	Scf00040	65370	87292		Terpene	Unknown
	Region 51.1	Scf00051	1	18148		Terpene	Unknown
	Region 94.1	Scf00094	2549	23508		Terpene	Squalestatin S1(40%)
	Region 429.1	Scf00429	1	270		Indole	Unknown
C. militaris	Region 37.1	AEVU01000037.g30	96433	100539	A-C	NRPS	Unknown
	Region 67.1	AEVU01000067.g44	143383	157733	P-A-A-P	NRPS	Emericellamide A/B(60%)
	Region 86.1	AEVU01000086.g65	206502	221056	A-P-A-P-C-P-C	NRPS	Unknown
	Region 136.1	AEVU01000136.g6	14827	18687	A-P-TE	NRPS	Unknown
	Region 160.1	AEVU01000160.g42	133839	147206	A-A-A-P-P	NRPS	Unknown
	Region 355.1	AEVU01000355.g43	114154	117954	C-A-C	NRPS	Unknown
	Region 358.1	AEVU01000358.g28	79375	84801	A-P-C-P-C	NRPS	Dimethylcoprogen(100%)
	Region 499.1	AEVU01000499.g4	6430	11645	A-P-C	NRPS	Unknown
	Region 535.1	AEVU01000535.g1	654	5198	A-C	NRPS	Unknown
	Region 122.1	AEVU01000122.g40	147727	152598	C-A-KS-AT	NRPS,T1PKS	Unknown
	Region 236.1	AEVU01000236.g15	45483	57314	KS-AT-DH-MT-KR-ACP-C-A-TE	NRPS,T1PKS	Unknown
	Region 346.1	AEVU01000346.g3	11212	23534	KS-AT--MT-KR-ACP-C-A-P-TE	NRPS,T1PKS	Phomasetin(85%)
	Region 444.1	AEVU01000444.g1	110	11106	KS-AT-DH-ER-KR-ACP-C-A-TE	NRPS,T1PKS	Unknown
	Region 468.1	AEVU01000468.g3	8314	20618	KS-AT-DH-MT-KR-ACP-C-A-P-TE	NRPS,T1PKS	Fumosorinone(83%)
	Region 185.1	AEVU01000185.g6	9834	16583	SAT-KS-AT-DH-PT-ACP-ACP-TE	NRPS,T1PKS	Unknown
	Region 474.1	AEVU01000474.g5	8426	15544	DIT-A-P-TE-MFS	NRPS,Other	Unknown
	Region 34.1	AEVU01000034.g7	15021	15021	C-A-C-MviM-WecE	NRPS,Other	Unknown
	Region 95.1	AEVU01000095.g3	5480	14937	SAT-KS-AT-PT-ACP-ACP-TE	NR-PKS	Viriditoxin(33%)

Region 509.1	AEVU01000509.g6	27540	35055	KS-AT-DH-MT-KR-ACP	PR-PKS	Unknown
Region 536.1	AEVU01000536.g10	37389	44845	KS-AT-DH-KR-ACP	PR-PKS	Unknown
Region 32.1	AEVU01000032.g7	14067	21690	KS-AT-DH-ER-KR-ACP	HR-PKS	1-nonadecene/(14Z)-1,14-nonadecadiene(100%)
Region 120.1	AEVU01000120.g23	89473	96664	KS-AT-DH-Ser-ER-KR-ACP	HR-PKS	Unknown
Region 67.2	AEVU01000067.g47	163793	172206	KS-AT-DH-MT-ER-KR-ACP	HR-PKS	Emericellamide A/B(60%)
Region 80.1	AEVU01000080.g7	15482	23025	KS-AT-ER-KR-ACP	HR-PKS	Unknown
Region 443.1	AEVU01000443.g2	4978	12615	KS-AT-ER-KR-ACP	HR-PKS	Unknown
Region 186.1	AEVU01000186.g9	26320	27195	TE	Other	Unknown
Region 219.1	AEVU01000219	84537	105157		indole	Unknown
Region 426.1	AEVU01000426	142836	164014		Terpene	Squalestatin S1(40%)
Region 544.1	AEVU01000544	117133	139142		Terpene	

Table S2. Overview of biosynthetic gene clusters in the genomes of the four studied fungi.

Species	NO of Cluster	PKS (Total)	Terpene	Hybrid NRPS-PKS	Hybrid NRPS-Other	NRPS	Other
C. pseudotenuipes (this study)	31	5	4	4	1	16	1
C. tenuipes (this study)	28	5	1	4	3	13	2
C. cicadae (ASM296887v1)	31	5	3	5	2	15	1
C. militaris (GCA_000225605.1)	29	8	2	6	2	9	2

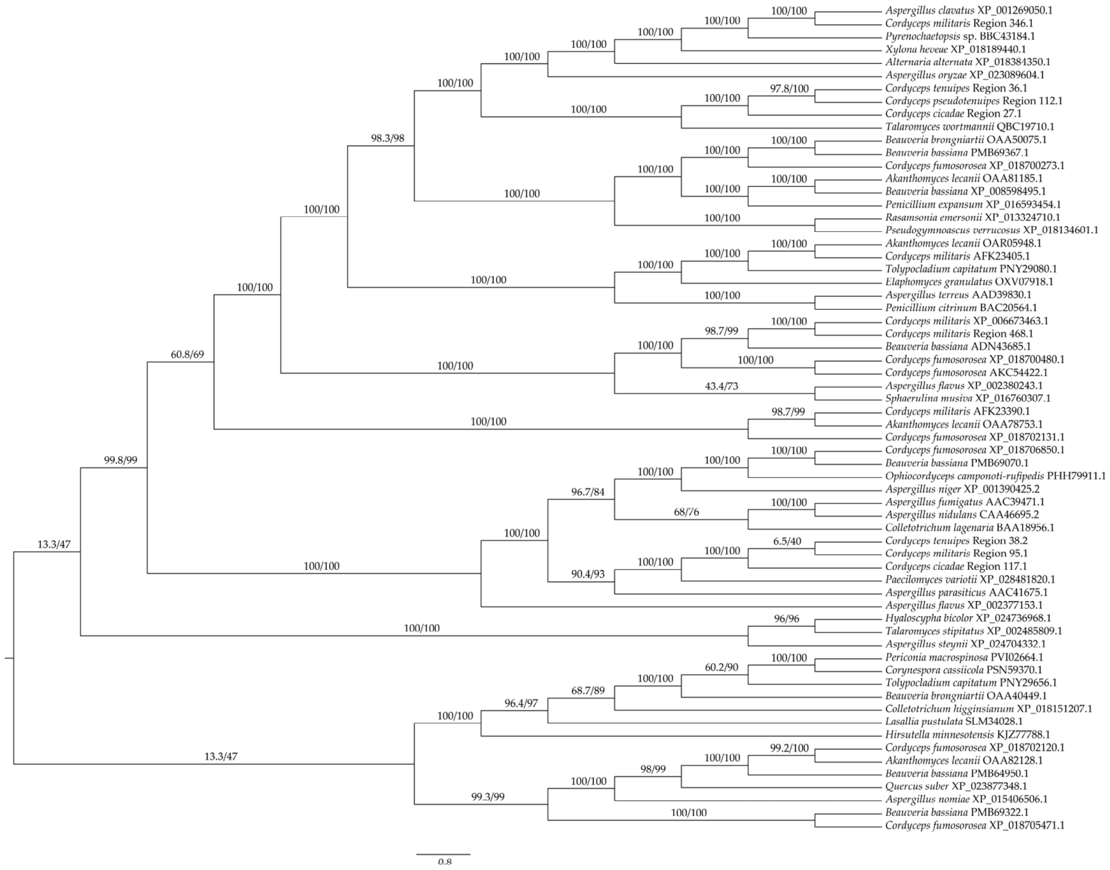

Figure S1. Clustering of the PKS/hybrid PKS-NRPS and other related fungal PKS/hybrid PKS-NRPS proteins in the four *Cordyceps* species.

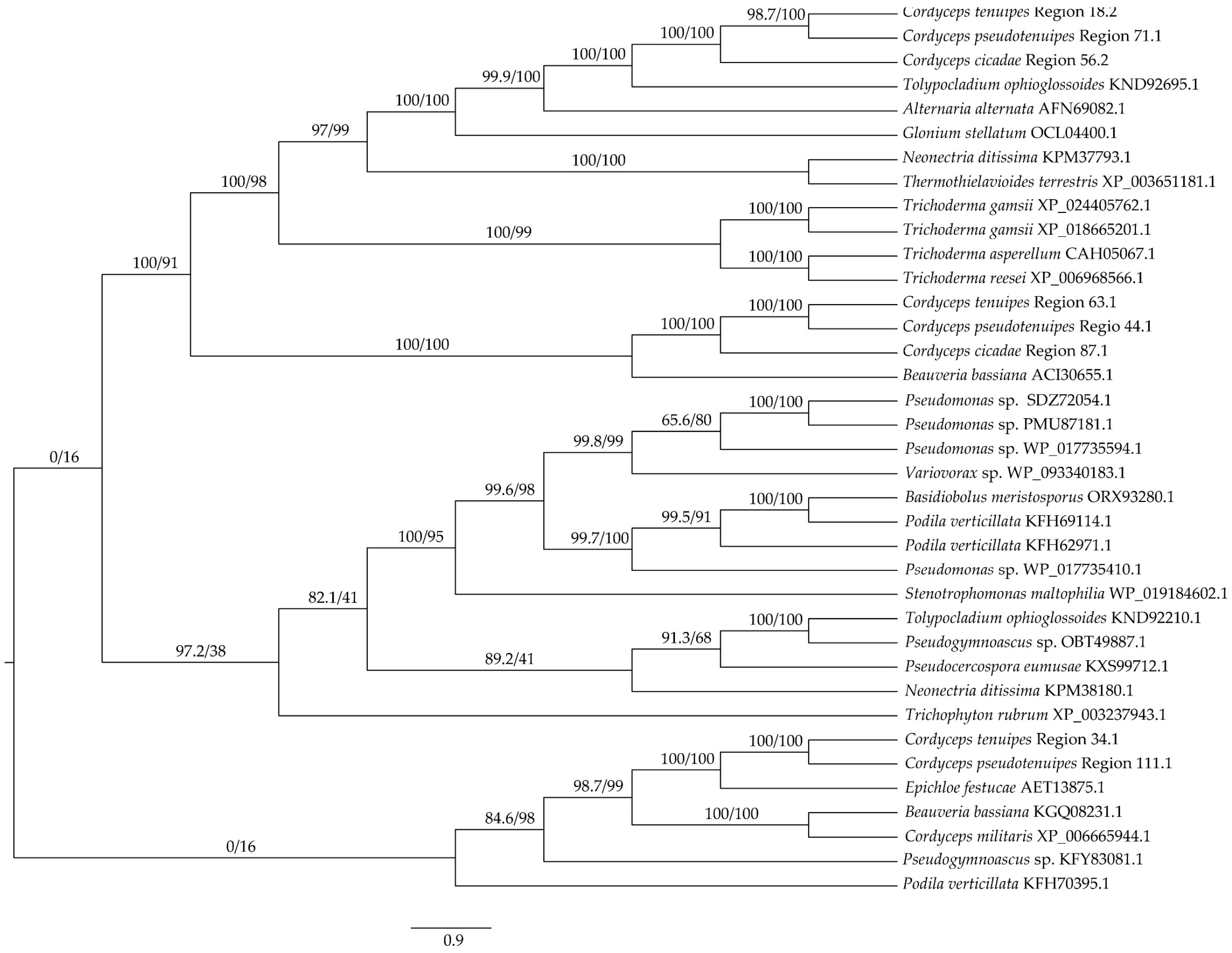

Figure S2. Clustering of NRPS and other fungal NRPS proteins in the four species of *Cordyceps*.
